# Secure Logistic Regression Based on Homomorphic Encryption: Design and Evaluation

**DOI:** 10.2196/medinform.8805

**Published:** 2018-04-17

**Authors:** Miran Kim, Yongsoo Song, Shuang Wang, Yuhou Xia, Xiaoqian Jiang

**Affiliations:** ^1^ Division of Biomedical Informatics University of California, San Diego San Diego, CA United States; ^2^ Department of Mathematical Sciences Seoul National University Seoul Republic Of Korea; ^3^ Department of Computer Science and Engineering University of California, San Diego San Diego, CA United States; ^4^ Department of Mathematics Princeton University Princeton, NJ United States

**Keywords:** homomorphic encryption, machine learning, logistic regression, gradient descent

## Abstract

**Background:**

Learning a model without accessing raw data has been an intriguing idea to security and machine learning researchers for years. In an ideal setting, we want to encrypt sensitive data to store them on a commercial cloud and run certain analyses without ever decrypting the data to preserve privacy. Homomorphic encryption technique is a promising candidate for secure data outsourcing, but it is a very challenging task to support real-world machine learning tasks. Existing frameworks can only handle simplified cases with low-degree polynomials such as linear means classifier and linear discriminative analysis.

**Objective:**

The goal of this study is to provide a practical support to the mainstream learning models (eg, logistic regression).

**Methods:**

We adapted a novel homomorphic encryption scheme optimized for real numbers computation. We devised (1) the least squares approximation of the logistic function for accuracy and efficiency (ie, reduce computation cost) and (2) new packing and parallelization techniques.

**Results:**

Using real-world datasets, we evaluated the performance of our model and demonstrated its feasibility in speed and memory consumption. For example, it took approximately 116 minutes to obtain the training model from the homomorphically encrypted Edinburgh dataset. In addition, it gives fairly accurate predictions on the testing dataset.

**Conclusions:**

We present the first homomorphically encrypted logistic regression outsourcing model based on the critical observation that the precision loss of classification models is sufficiently small so that the decision plan stays still.

## Introduction

Biomedical data are highly sensitive and often contain important personal information about individuals. In the United States, health care data sharing is protected by the Health Insurance Portability and Accountability Act [1], whereas biomedical research practitioners are covered under federal regulation governing the “Common Rule,” a federal policy that protects people who volunteer for federally funded research studies [2]. These policies set high standards on the protection of biomedical data and violations will lead to financial penalties and lost reputation. On the other hand, cloud computing, which significantly simplifies information technology environments, is the trend for data management and analysis. According to a recent study by Microsoft, nearly a third of organizations work with four or more cloud vendors [3]. The privacy concern, therefore, becomes a major hurdle for medical institutions to outsource data and computation to the commercial cloud. It is imperative to develop advanced mechanisms to assure the confidentiality of data to support secure analysis in the cloud environment.

An intuitive solution is to train a model without accessing the data and only obtain the estimated model parameters in a global manner. Assuming summary statistics can be shared, this can be done in a joint manner and we have developed the “grid logistic regression” [4-6] to show the feasibility of estimating the global parameters from distributed sources (eg, by only exchanging gradients and Hessian matrices). However, there are still vulnerabilities in sharing even the summary statistics; for example, the difference in mean age between a cohort of *n* patients and another cohort of *n* –1 overlapped patients can reveal the actual age of a single patient.

**Figure 1:**
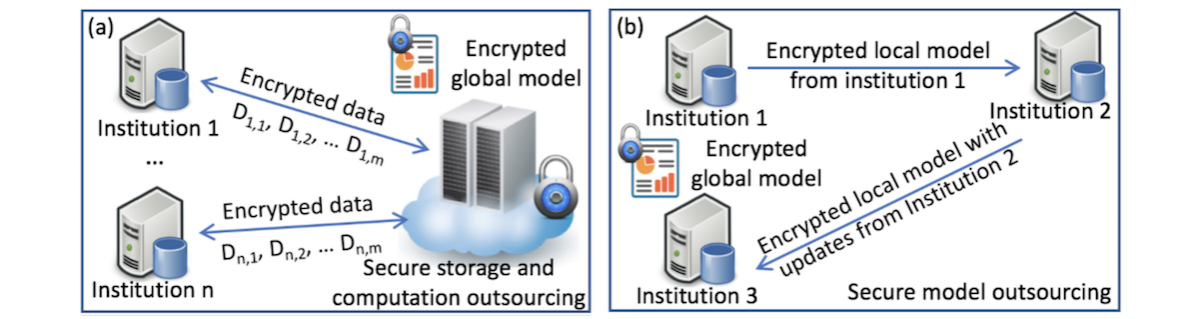
Two secure models: (a) secure storage and computation outsourcing and (b) secure model outsourcing.

Many medical decision-making systems rely on the logistic regression model [7-9]. However, to use them appropriately, we need to provide a sufficient sample, which requires a sample size calculation. Peduzzi et al [10] suggested a simple guideline for a minimum number of cases to include in the study: let *p* be the smallest of the proportions of negative or positive cases in the population and *k* the number of covariates (the number of independent variables), then the minimum number of cases to include is *N* = 10 · *k* / *p*. For example, one has three covariates to be included in the model and the proportion of positive cases in the population is 0.2 (20%). The minimum number of cases required is 10 · 3 / 0.20 = 150. For rare disease studies with many variables, it is even harder to collect enough samples from a single institution to meet this goal. We need to circumvent the privacy barriers to feed the model with more samples from different sources. As shown in Figure 1, homomorphic encryption techniques can support typical secure computations (eg, secure outsourcing and secure multiparty computation) and mitigate the privacy risks by allowing all computation to be done in the encrypted format.

Graepel et al [11] shed light on machine learning with homomorphically encrypted data. The article discussed scenarios that are appropriate and inappropriate to exercise machine learning with homomorphic encryption techniques. The authors provided two examples: linear means classifier and linear discriminative analysis, which can be achieved by using low-degree polynomials in homomorphic encryption. However, these simple parametric models do not handle complex datasets well and they do not represent the mainstream machine learning technologies used in biomedical research [12,13]. Additional work was carried out by Bos et al [14] to demonstrate the feasibility of making a prediction on encrypted medical data in Microsoft’s Azure cloud. However, instead of learning from the data, this model only makes predictions using learned logistic regression models in a privacy-preserving manner. Similarly, a more recent work called CryptoNets applied trained neural networks to encrypted data only for the evaluation purpose [15]. Related works are summarized in Table 1.

In the current literature, most similar to our work are Aono et al [16] and Mohassel et al [17], but they are also very different from ours in assumptions and methods. Aono et al introduced an approximation to convert the likelihood function into a low-degree polynomial and used an additive homomorphic encryption to aggregate some intermediary statistics [16]. However, their scenario relies on the client to decrypt these intermediary statistics so that it can minimize the parameters locally. This is not a completely secure outsourcing scenario as ours, which works on encrypted data to obtain encrypted parameters without any client involvement. Mohassel et al developed secure two-party computation protocols to conduct the stochastic gradient descent for solving logistic regression and neural network problems [17].

**Table 1:** Research works in secure analysis.

Reference	Problem	Techniques
Graepel et al [11]	Linear means classifier/discriminative analysis	Homomorphic encryption
Bos et al [14]	Prediction using learned logistic regression model	Homomorphic encryption
Dowlin et al [15]	Prediction using learned neural networks	Homomorphic encryption
Aono et al [16]	Logistic regression	Additive homomorphic encryption
Mohassel et al [17]	Logistic regression	Multiparty computation
This work	Logistic regression	Homomorphic encryption

This method takes a completely different approach (garbled circuit and secret sharing vs homomorphic encryption) and the assumptions are widely different from ours (secure multiparty computation vs secure outsourcing). There are several prominent challenges related to scalability and efficiency. Traditional methods cannot handle many iterations of multiplications, which leads to a deep circuit and exponential growth in computational cost and storage size of the ciphertext. On the other hand, it is a nontrivial task to approximate certain critical functions in machine learning models using only low-degree polynomials. Naive approximation may lead to big errors and makes the solutions intractable. Our framework proposes novel methods to handle these challenges and makes it possible to learn a logistic regression model on encrypted data based completely on homomorphic encryption.

## Methods

### Logistic Regression

Logistic regression is a widely used learning model in biomedicine [13]. Data for supervised learning consist of pairs (*x*_*i*_, *y*_*i*_) of a vector of covariates *x*_*i*_ = (*x*_*i* 1_,..., *x*_*i* d_) and a class label *y*_*i*_ for *i* = 1,..., *n*. We assume that *y*_*i*_ = 1 / –1 for binary classification. The model looks like:

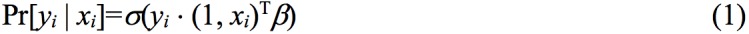



for the sigmoid function *σ*(*x*) = 1 / [1 + exp(–*x*)] where *β* = (*β*_0_, *β*_1_,..., *β*_*d*_) are the model parameters to be estimated. Training methods of logistic regression aim to find the optimal parameters *β*, which minimizes the cost (negative log-likelihood)

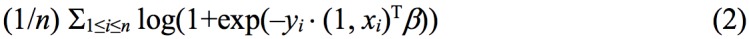



### Homomorphic Encryption for Approximate Arithmetic

Homomorphic encryption is an encryption technique that allows computations on ciphertexts and generates encrypted results that match those of plaintext computation. We adopted a special cryptosystem developed by Cheon et al [18], which supports an approximate arithmetic of encrypted messages. Different from existing methods, this cryptosystem trades precision for efficiency so that the size of parameters does not grow too large (thus computationally feasible). Interested readers can refer to Multimedia Appendix 1 for more details. The cryptosystem supports key generation, encryption, decryption, addition, and multiplication operations. It also supports message packing and rotation, which are important to parallelize similar tasks.

A unique property of this cryptosystem is the following rescaling procedure, which plays an important role in controlling the magnitude of messages and, therefore, achieving the efficiency of approximate computation. The rescaling procedure coverts an encryption *ct* of a message *m* with a ciphertext modulus *q* into an encryption *ct'* of *r*^-1^ ⋅ m under the same secret key but a smaller modulus *q'* = *r*^-1^ ⋅ *q*, in which *r* is a scaling factor. We denote the output ciphertext by RS(*ct*; *r*). It enables us to round the message and reduce the size of significand by removing some inaccurate least significant bits as in the floating-point arithmetic. Informally, we will say that the input ciphertext modulus is reduced by log *r* bits after this procedure where the binary logarithm will be simply denoted by log(⋅).

### Least Squares Approximation of the Sigmoid Function

Unlike linear regression, logistic regression does not have a closed-form solution in most cases. As a result, we need to use nonlinear optimization methods to find the maximum likelihood estimators of the regression parameters. The Newton-Raphson [19] and the gradient descent [20] are the most commonly used methods for training. Because the Newton-Raphson method involves matrix inversion and most homomorphic encryption schemes do not naturally support division or matrix inversion, it is difficult to evaluate the method with homomorphic encryption schemes. On the other hand, gradient descent does not require the division operation and, therefore, it is a better candidate for homomorphically encrypted logistic regression. Thus, we choose the gradient descent algorithm as the training method for logistic regression.

Let (*x*_*i*_, *y*_*i*_) be the supervised learning samples for *i* = 1,..., *n*. If we write *z*_*i*_ = *y*_i_ ⋅ (1, *x*_*i*_), the cost function for logistic regression is defined by:

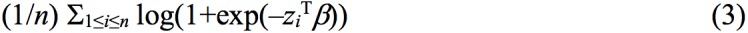



Its gradient with respect to *β* is computed by –1 / *n*Σ_1≤*i*__≤__*n*_*σ* (–*z*_*i*_^T^*β*) ⋅ *z*_*i*_. To find a local minimum point, the gradient descent method updates the regression parameters using the following formula until *β* converges:

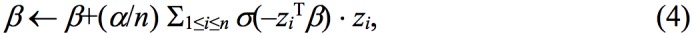



where *α* is the learning rate.

Although the gradient descent method seems better suited than other training methods for homomorphic evaluation, some technical problems remain for implementation. In the preceding update formula, the sigmoid function is the biggest obstacle for evaluation, since the existing homomorphic encryption schemes only allow evaluation of polynomial functions. Hence, the Taylor polynomials *T*_d_(*x*) = Σ_0≤*k≤d*_(*f*^ (k)^(0) / *k*!) ⋅ *x*^*k*^ have been commonly used for approximation of the sigmoid function [14,17]:

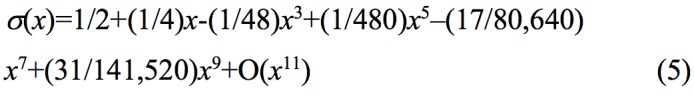



**Figure 2:**
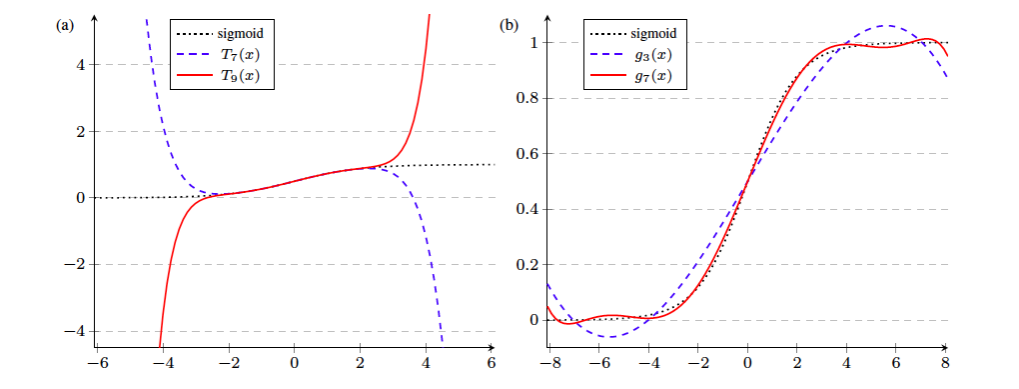
Graphs of (a) sigmoid function and Taylor polynomials and (b) sigmoid function and least squares approximations.

However, we observed the input values *z*_*i*_^T^*β* of the sigmoid function during iterations on real-world datasets and concluded that the Taylor polynomial *T*_9_(*x*) of degree 9 is still not enough to obtain the desired accuracy (see Figure 2a). The size of error grows rapidly as | *x* | increases. For instance, we have *T*_9_(4) ≈ 4.44, *T*_9_(6) ≈ 31.23, and *T*_9_(8) ≈ 138.12. In addition, we have to use a higher degree Taylor polynomial to guarantee the accuracy of regression, but it requires too many homomorphic multiplications to be practically implemented. In summary, the Taylor polynomial is not a good candidate for approximation because it is a local approximation near a certain point. Therefore, we adopted a global approximation method that minimizes the mean squared error (MSE). For an integrable function *f*, its mean square over an interval *I* is defined by (1 / | *I* |) ∫_*I*_ *f* (*x*)^2^d*x*, where | *I* | denotes the length of *I*. The least squares method aims to find a polynomial *g*(*x*) of degree *d* which minimizes the MSE (1 / | *I* |) ∫_*I*_ (*f*(*x*) – *g*(*x*))^2^d*x*. The least squares approximation has a closed formula that can be efficiently calculated using linear algebra.

In our implementation, we used the degree 3 and 7 least squares approximations of the sigmoid function over the interval [–8,8], which contains all of the input values (– *z*_*i*_^T^*β*) during iterations. The least squares polynomials are computed as:

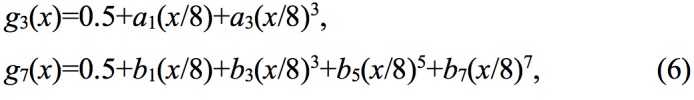



where the coefficients vectors are (*a*_1_, *a*_3_) ≈ (1.20096,–0.81562) and (*b*_1_, *b*_3_, *b*_5_, *b*_7_) ≈ (1.73496,–4.19407, 5.43402,–2.50739). The degree 3 least squares approximation requires a smaller depth for evaluation, whereas the degree 7 polynomial has a better precision (see Figure 2b).

### Homomorphic Evaluation of Gradient Descent Algorithm

We will describe how to encode data and explain how to analyze logistic regression on encrypted data. To speed up the computation, we will use the packing mechanism to batch *n* slots and perform *n* evaluations in parallel, where *n* is the number of training data samples.

We start with a useful aggregation operation across plaintext slots from the literature [21-23]. Specifically, given a ciphertext representing a plaintext vector (*m*_1_, *m*_2_,..., *m*_k_), we introduce an algorithm (denoted by AllSum) that generates a ciphertext representing a value of Σ_1≤i__≤__k_*m*_i_ in each plaintext slot. Assume that *k* is chosen as a power-of-two integer. The cyclic rotation by one unit produces a ciphertext encrypting the plaintext vector (*m*_2_,..., *m*_k_, *m*_1_). Then an encryption of the vector (*m*_1_ + *m*_2_, *m*_2_ + *m*_3_,..., *m*_k_ + *m*_1_) is obtained by adding the original ciphertext. We repeatedly apply this method (log *k* – 1) times with a rotation by a power of two, which generates the desired ciphertext; that is, every plaintext slot contains the same value of Σ_1≤i__≤__k_*m*_i_. The AllSum algorithm is explicitly described in Textbox 1.

Let us assume that we are given *n* training data samples *z*_i_ with (*d* +1) features. As mentioned before, our goal is to securely evaluate the following arithmetic circuit:

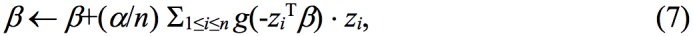



where *g*(*x*) denotes the approximating polynomial of the sigmoid function chosen in the previous subsection. We set the initial *β* parameters as the zero vector for simplicity.

Because our cryptosystem only supports integer computation, all the elements are scaled by a factor of an integer *p* and then converted into the nearest integers for quantization. The client first receives the ciphertexts encrypting the vector (*p* · *z*_*i*_) from *n* users, and then compromises them to obtain (*d* + 1) ciphertexts *ct.z*_*i*_ for all *j*  = 0,1,..., *d*, each of which encrypts the vector *p* ⋅ (*z*_*1j*_,..., *z*_*nj*_) of the *j*-th attributes using batching technique. If *n* is not a power of two, the plaintext slots are zero padded so that the number of slots divides *N* / 2. Finally, these resulting ciphertexts (*ct.z*_0_,..., *ct.z*_*d*_) are sent to the server for the computation of gradient descent.

**The AllSum algorithm.**

0: **Inputs:** ciphertext *ct* encrypting plaintext vector (*m*_1_, *m*_2_,..., *m*_k_).

1: **For** *i* = 0,1,..., log *k* –1** do**

2: Compute *ct* ←Add(*ct*, Rot(*ct*;2^*i*^))

3: **end for**

4: **Outputs:** ciphertext *ct* encrypting Σ_1≤ *i≤k*_*m*_*i*_ in each plaintext slot

**Secure logistic regression algorithm.**

0: **Inputs:** Ciphertexts { *ct.z*_j_}_0≤j__≤__d_, a polynomial *g*(*x*), a number of iterations *IterNum*

1: **For** *j* = 0,1,…, *d* **do**

2: *ct.beta*_*j*_ ←**0**

3: **end for**

4: **For ***k* = 1,2,…, *IterNum* **do**

5: *ct.ip* ←RS(∑_0≤ *j≤d*_Mult(*ct.beta*_*j*_, *ct.z*_*j*_); *p*)

6: *ct.g* ←PolyEval(– *ct.ip*, ⌊ *p* · *g*(*x*)⌉)

7: **For** *j* = 0,1,…, *d* **do**

8: *ct.grad*_*j*_ ←RS(Mult(*ct.g*, *ct.z*_*j*_); *p*)

9: *ct.grad*_*j*_ ←RS(AllSum(*ct.grad*_*j*_); ⌊*n* / *α*⌉)

10: *ct.beta*_*j*_ ←Add(*ct.beta*_*j*_, *ct.grad*_*j*_)

11: **end for**

12: **end for**

13: **Outputs:** Ciphertexts { *ct.beta*_> *j*_}_0≤ *j≤d*_

The public server generates the initial ciphertexts (*ct.beta*_*0*_,..., *ct.beta*_*d*_) as zero polynomials in R_*q*_ (the residue ring of R = Z[X] / (X^*N*^ + 1) modulo an integer *q*). At each iteration, it performs a homomorphic multiplication of ciphertexts *ct.beta*_*j*_ and *ct.z*_*j*_, and outputs a ciphertext encrypting the plaintext vector *p*^2^ ⋅ (*z*_*1j*_^T^*β*_*j*_,..., *z*_*nj*_^T^*β*_*j*_) for all *j* = 0,..., *d*. Then it aggregates the ciphertexts and performs the rescaling operation with a scaling factor of *p* to manipulate the size of plaintext, returning a ciphertext *ct.ip* that represents a plaintext vector approximating to *p* ⋅ (*z*_*1*_^T^*β*,..., *z*_n_^T^*β*).

For the evaluation of the least squares polynomial *g*(*x*) at (–*z*_*i*_^T^*β*), we adapt the polynomial evaluation algorithm, denoted by PolyEval(⋅), suggested in [18]. Each coefficient of the polynomial should be scaled by a factor of p to be transformed into an integral polynomial. The output ciphertext *ct.g* contains *p* ⋅ *g*(–*z*_*i*_^T^*β*) in the *i*-th slot. Finally, the server performs a homomorphic multiplication of the ciphertexts *ct.g* and *ct.z*_*j*_, AllSum procedure, and rescaling by a factor of ⌊ *n / α* ⌉ (nearest integer to *n / α*). These procedures generate ciphertexts *ct.grad*_0_,..., *ct.grad*_*d*_ corresponding to the entries of the gradient vector weighted by the learning rate and the sample size. Then it only needs to perform an addition with the model parameters *β* and the gradient vector over encryption, which yields a new ciphertext *ct.beta*_*j*_ that encrypts an approximation of the *j*-th scaled value of the gradient update in Equation 7. Our secure logistic regression algorithm is described in Textbox 2.

Our solution can compute the gradient descent algorithm securely; however, its direct implementation is not efficient and requires a total ciphertext modulus of log *p* ⋅ (⌈log deg(*g*)⌉ + 3) + ⌈log (*n* / *α*)⌉ bits at each iteration, where ⌈ *x* ⌉ denotes the smallest integer that is not less than *x*. We further optimized this algorithm by manipulating the arithmetic circuit for the update term (*α* / *n*)Σ_1≤*i*≤n_ *g*(–*z*_*i*_^T^*β*) ⋅ *z*_*i*_ and could reduce the ciphertext modulus to 3 ⋅ log *p* + ⌈log (*n* / 4*α*)⌉ bits or 4 · log *p* + ⌈log (*n* / 4*α*)⌉ bits when *g*(*x*) = *g*_3_(*x*) or *g*(*x*) = *g*_7_(*x*), respectively. Interested readers can refer to Multimedia Appendix 2 for more details.

## Results

### Implementation Details

All experiments were performed on an Intel Xeon running at 2.3 GHz processor with 16 cores and 64 GB of RAM, which is an m4.4xlarge AWS EC2 instance. In our implementation, we used a variant of a fixed-point homomorphic encryption scheme of Cheon et al [18,24] with C++-based Shoup’s Number Theory Library [25]. Our implementation is publicly available at GitHub [26].

### Datasets

We developed our approximation algorithm using the Myocardial Infarction dataset from Edinburgh [27]. The others were obtained from Low Birth Weight Study, Nhanes III, Prostate Cancer Study, and Umaru Impact Study datasets [28-31]. All these datasets have a single binary outcome variable, which can be readily used to train binary classifiers such as logistic regression. Table 2 illustrates the datasets with the number of observations (rows) and the number of features (columns), respectively. We utilized five-fold cross-validation that randomly partitions the original datasets into five folds with the approximately equal size; we used four subsets for learning (with the learning rate *α* ≈ 1) and one subset for testing the trained model..

### Parameters and Timings for the Homomorphic Encryption Scheme

We assumed that all inputs had log *p* = 28 bits of precision and set the bit length of the output ciphertext modulus as log *q*_0_ = log *p* + 10. As discussed previously, when evaluating the gradient descent algorithm with *g*(*x*) = *g*_7_(*x*), a ciphertext modulus is reduced more than *g*(*x*) = *g*_3_(*x*) at each iteration. Thus, we set the number of iterations as *IterNum* = 25 (resp *IterNum* = 20) when *g*(*x*) = *g*_3_(*x*) (resp. *g*(*x*) = *g*_7_(*x*)) to take an initial ciphertext modulus of similar size. We could actually obtain the approximate bit length of fresh ciphertext modulus log *q* around 2204 to 2406. The parameter set provides 80 bits of security (see Multimedia Appendix 3 for more details). Because all the computations were performed on encrypted data, the security against a semi-honest adversary follows from the semantic security of the underlying homomorphic encryption scheme. For this setting, the size of the public key and a freshly encrypted ciphertext is 75 MB. The key generation takes approximately 56 to 58 seconds and the encryption takes approximately 1.1 to 1.3 seconds.

In Table 3, we evaluated our models performance based on average running time (encryption, evaluation, and decryption) and storage (encrypted dataset size) in each fold.

**Figure 3:**
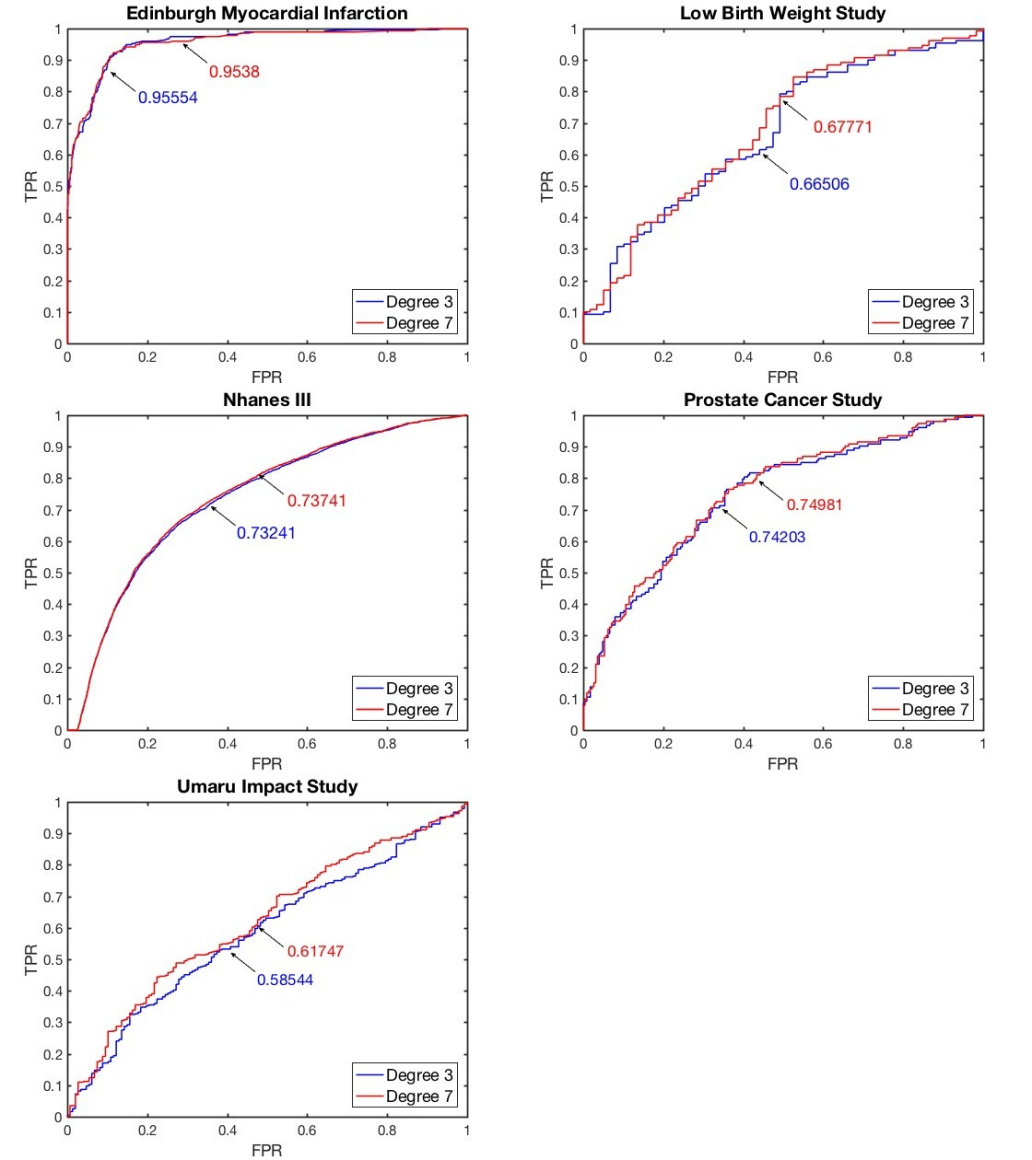
Average AUC of encrypted logistic regression. FPR: false positive rate; TPR: true positive rate.

We used a popular metric, area under the receiver operating characteristic curve (AUC), to measure the model’s classification performance when the true positive rate was plotted against the false positive rate at various thresholds. Figure 3 plots the average AUC values from five-fold cross-validation for datasets. The program was implemented by MATLAB 2017a.

We can converge to the optimum within a small number of iterations (20~25), which makes it very promising to train a homomorphically encrypted logistic regression model and mitigate the privacy concerns.

In Table 4, we compared the produced models using our encrypted approach and unencrypted logistic regression. In the unencrypted cases, we used the original sigmoid function on the same training dataset with the same iteration numbers as the encrypted cases. For discrimination, we calculated the accuracy (%), which is defined by the percentage of the correct predictions on the testing dataset. For a more accurate comparison, we used the MSE that measures the average of the squares of the errors. We could also normalize it by dividing by the average of the squares of the (unencrypted) model parameters, called a normalized mean squared error (NMSE).

**Table 2:** Description of datasets.

Dataset	Number of observations	Number of features
Edinburgh Myocardial Infarction	1253	10
Low Birth Weight Study	189	10
Nhanes III	15,649	16
Prostate Cancer Study	379	10
Umaru Impact Study	575	9

**Table 3:** Experiment results of our homomorphic encryption-based logistic regression algorithm

Dataset and degree of *g*(*x*)		Encryption (sec)	Evaluation (min)	Decryption (sec)	Storage (GB)
**Edinburgh Myocardial Infarction**					
	3	12	131	6.3	0.69
	7	12	116	6.0	0.71
**Low Birth Weight Study**					
	3	11	101	4.9	0.67
	7	11	100	4.5	0.70
**Nhanes III**					
	3	21	265	12	1.15
	7	21	240	13	1.17
**Prostate Cancer Study**					
	3	11	119	4.4	0.68
	7	11	100	4.5	0.70
**Umaru Impact Study**					
	3	10	109	5.1	0.61
	7	10	94	4.3	0.63

**Table 4:** Comparison of encrypted/unencrypted logistic regression. AUC: area under the receiver operating characteristic curve. MSE: mean squared error; NMSE: normalized mean squared error.

Dataset and iteration number		Degree of *g*(*x*)	Our homomorphic encryption-based logistic regression		Unencrypted logistic regression		MSE	NMSE
			Accuracy	AUC	Accuracy	AUC		
**Edinburgh Myocardial Infarction**								
	25	3	86.03%	0.956	88.43%	0.956	0.0259	0.0261
	20	7	86.19%	0.954	86.19%	0.954	0.0007	0.0012
**Low Birth Weight Study**								
	25	3	69.30%	0.665	68.25%	0.668	0.0083	0.0698
	20	7	69.29%	0.678	69.29%	0.678	0.0003	0.0049
**Nhanes III**								
	25	3	79.23%	0.732	79.26%	0.751	0.0033	0.0269
	20	7	79.23%	0.737	79.23%	0.737	0.0002	0.0034
**Prostate Cancer Study**							
	25	3	68.85%	0.742	68.86%	0.750	0.0085	0.0449
	20	7	69.12%	0.750	69.12%	0.752	0.0002	0.0018
**Umaru Impact Study**						
	25	3	74.43%	0.585	74.43%	0.587	0.0074	0.0829
	20	7	75.43%	0.617	74.43%	0.619	0.0004	0.0077

## Discussion

### Principal Findings

Our implementation shows that the evaluation of the gradient descent algorithm with the degree 7 least squares polynomial yields better accuracy and AUC than degree 3. It is quite close to the unencrypted result of logistic regression using the original sigmoid function with the same number of iterations; for example, on the training model of Edinburgh dataset, we could obtain the model parameters *β* as follows:

(–1.7086, 0.0768, 0.1119, 0.3209, 1.2033,

0.3684, 0.9756, 0.2020, 0.2259, –0.1641),

which can reach 86.19% accuracy and 0.954 AUC on the testing dataset. When using the sigmoid function on the same training dataset, the model parameters *β* are

(–1.6308, 0.0776, 0.1097, 0.3155, 1.1809,

0.3651, 0.9599, 0.2083, 0.2298, –0.1490),

which give the same accuracy and AUC. On the other hand, as shown in Table 4, the MSE and NMSE values of degree 7 are closer to zero which inspires us that the polynomial approximation of that degree is fairly accurate for logistic regression.

One of the inherent properties of our underlying homomorphic encryption scheme is that the inserted errors for security may increase after some homomorphic operations. Hence, the size of error and the precision loss should be discussed carefully to guarantee the correctness of the resulting value. On the other hand, the gradient descent method has a property of negative feedback on computational error. Because we use the gradient at the current weight vector β to move it closer to the optimal point of minimized cost, the effect of noise disappears after some iterations. Therefore, there is no need to manage the precision of messages to confirm the correctness of resulting value because the noises are not amplified during evaluation. In our experimentation on the Edinburgh dataset, for instance, the difference between the model parameters obtained from encrypted/unencrypted evaluations was less than 2^-11^. This means that we can precisely compute at least most significant 11 bits after the radix point of the model parameters and this approximate vector is accurate enough to achieve a good performance in testing data samples.

### Limitations

There are still a number of limitations in the application of our evaluation model to an arbitrary dataset. First, the use of homomorphic encryption yields the overheads in computation and storage. The size of the dataset should be limited for practical evaluation, but this is not a big problem because there have been significant improvements in the existing homomorphic encryption schemes. The development of homomorphic encryption technology will achieve much better practical performance in our protocol.

Another issue arises from the polynomial approximation. We suggested the least squares method on a certain interval [–8,8], but the precision of the result can increase by managing approximation error from wider range inputs. Finally, our model is based on fixed hyperparameters that should be decided before starting of the evaluation. It would be highly beneficial if we could detect convergence of the loss function in the training process and support early stop instead.

### Conclusions

This paper presents the first effective methodology to evaluate the learning phase of logistic regression using the gradient descent method based on homomorphic encryption. We have demonstrated the capability of our model across the experiments with different biological datasets. In particular, our solution can be applied to a large-scale dataset, which shows the feasibility of our approach.

## Appendix

Multimedia Appendix 1 Homomorphic encryption for approximate arithmetic.

Multimedia Appendix 2 Further optimization of secure logistic regression algorithm.

Multimedia Appendix 3 How to set parameters.

## Abbreviations

AUC: area under the curve
MSE: mean squared error
NMSE: normalized mean squared error
