# Supplementary material for: Secure Logistic Regression Based on Homomorphic Encryption: Design and Evaluation
Source: JMIR Med Inform. 2018 Apr 17;6(2):e19. doi: 10.2196/medinform.8805 (PMC5930176; doi:10.2196/medinform.8805)
Supplement: Multimedia Appendix 1 [file medinform_v6i2e19_app1.pdf]

Homomorphic Encryption for Approximate Arithmetic

Homomorphic encryption is a cryptosystem that supports arithmetic operations between ciphertexts. This technology has great potentials in real-world applications since it allows us to securely outsource expensive computation on public (untrusted) server. The security of practical homomorphic encryption schemes is based on the hardness of ring learning with errors problem. The inherent limitation of these constructions is that they only support the arithmetic operations over modular spaces so the required size of parameter for real number operations (ie, no modular reduction over plaintext space) is too large to be practically implemented. On the other hand, real-world applications such as statistical testing, neural networks, and machine learning do not require absolute precision and they are all to a certain degree approximate.

Recently, Cheon et al presented a new homomorphic encryption scheme which supports an approximate arithmetic of encrypted messages [18]. The main idea is to consider an encryption noise (on the ciphertext for security) as a part of computation error occurring during approximate arithmetic. In other words, given a secret key *sk*, an encryption *ct* of a plaintext *m* satisfies the equation $\langle ct,sk\rangle = m+e \pmod q$ for some small error e.

We suppose that the underlying HE scheme is based on the RLWE assumption over the cyclotomic ring $R=Z[X]/(X^N+1)$ for being a power of two. Let us denote by $[\cdot]_q$ the reduction mod q into the interval $(-q/2,q/2]\cap Z$ of the integer. We write $R_q= R/qR$ for the residue ring of R modulo an integer q. The following is a description of the homomorphic encryption scheme [18].

- ParamsGen(λ): Given the security parameter λ, choose a power-of-two integer N, a modulus $Q=q^2$ and a discrete Gaussian distribution χ. The RLWE problem of parameter (N, Q, χ) should achieve at least λ bits of security level of cryptosystem. Output *params* $\leftarrow (N, q, \chi)$.
- KeyGen(*params*): Generate a polynomial $s(X) \in R$ by sampling its coefficient vector randomly from a sparse distribution on $\{0,1,-1\}^N$ and set the secret key as $sk \leftarrow (1, s(X))$. Sample a polynomial $a(X)$ uniformly at random from R_q and an error polynomial $e(X)$ from χ. Set the public key as $pk\leftarrow(b(X), a(X)) \in R_q\times R_q$ where $b(X) \leftarrow -a(X)\cdot s(X)+e(X) \pmod q$. Let $s'(X) \leftarrow s(X)^2$. Sample a polynomial $a'(X)$ uniformly at random from R_Q and $e'(X)$ from χ. Set the evaluation key as $evk \leftarrow (b'(X), a'(X))\in R_Q\times R_Q$ where $b'(X) \leftarrow -a'(X)\cdot s(X)+e'(X) +q\cdot s'(X) \pmod Q$.

- $\mathrm{Enc}_{pk}(m)$: Sample a small polynomial $v(\mathrm{X})$ (with 0,1,-1 coefficients) and two error polynomials $e_0(\mathrm{X})$, $e_1(\mathrm{X})$ from χ. Output the ciphertext $ct \leftarrow v(\mathrm{X}) \cdot pk + (m+e_0(\mathrm{X}), e_1(\mathrm{X})) \in \mathrm{R}_q \times \mathrm{R}_q$.
- $\mathrm{Dec}_{sk}(ct)$: For $ct = (c_0(\mathrm{X}), c_1(\mathrm{X}))$, output $m \leftarrow c_0(\mathrm{X}) + c_1(\mathrm{X}) \cdot s(\mathrm{X}) \pmod{q}$.
- $\mathrm{Add}(ct, ct')$: Add two ciphertexts and output $ct_{\mathrm{add}} \leftarrow ct + ct' \pmod{q}$.
- $\mathrm{Mult}(ct, ct')$: For two ciphertexts $ct = (c_0(\mathrm{X}), c_1(\mathrm{X}))$ and $ct'=(c_0'(\mathrm{X}), c_1'(\mathrm{X}))$, let $(d_0(\mathrm{X}), d_1(\mathrm{X}), d_2(\mathrm{X})) = (c_0(\mathrm{X}) \cdot c_0'(\mathrm{X}),\ c_0(\mathrm{X}) \cdot c_1'(\mathrm{X}) + c_1(\mathrm{X}) \cdot c_0'(\mathrm{X}),\ c_1(\mathrm{X}) \cdot c_1'(\mathrm{X})) \pmod{q}$. Output the ciphertext $ct_{\mathrm{mult}} \leftarrow (d_0(\mathrm{X}), d_1(\mathrm{X})) + \lfloor q^{-1} \cdot d_2(\mathrm{X}) \cdot evk \rceil \pmod{q}$.

The native plaintext space is simply represented as the set of polynomials in R with coefficient less than q, but it can be understood as a vector of $(N/2)$-dimensional complex vector using the encoding/decoding method described in [18] (each factor is called a plaintext slot). This technique enables us to parallelize both space and computation time. Addition and multiplication in R correspond the component-wise addition and multiplication on plaintext slots. In addition, we could get a shift of the plaintext slots, that is, if ct encrypts a plaintext vector $(m_1,m_2,\ldots,m_k)$, we could obtain the encryption of the shifted vector $(m_2,\ldots,m_k,m_1)$.

This homomorphic encryption scheme has its own distinct and unique characteristics represented by the following rescaling procedure.

- $\mathrm{RS}(ct; r)$. For a ciphertext $ct \in \mathrm{R}_q \times \mathrm{R}_q$ and $r>0$, output the ciphertext $ct' \leftarrow \lfloor r^{-1} \cdot ct \rceil \in \mathrm{R}_{q'} \times \mathrm{R}_{q'}$ for $q'=r^{-1} \cdot q$.
