# Supplementary material for: Secure Logistic Regression Based on Homomorphic Encryption: Design and Evaluation
Source: JMIR Med Inform. 2018 Apr 17;6(2):e19. doi: 10.2196/medinform.8805 (PMC5930176; doi:10.2196/medinform.8805)

Further Optimization of Secure Logistic Regression Algorithm

Secure logistic regression algorithm in Textbox 2 reduces the ciphertext modulus to log p ·(⌈log deg (g)⌉+3) + ⌈log(n/α)⌉ bits to update the encryption of β at each iteration. For the efficient implementation, we employ some techniques to reduce the number of consumed bits during the evaluation procedure. We express the evaluation circuit as follows:

$$\beta \leftarrow \beta + (4\alpha/n)\ \Sigma_{1\le i\le n}(z_i/8) - (4\alpha/n)\ \Sigma_{1\le i\le n}(2g(z_i^{\mathrm{T}}\beta)-1)\cdot(z_i/8). \quad \text{(A-1)}$$

If the client generates encryptions of $p\cdot(z_i/8)$ instead of $p\cdot z_i$, the required bit length of ciphertext modulus per iteration can be reduced. On the other hand, the server uses a pre-computation step to reduce the complexity of the update equation: it performs AllSum procedure and applies the rescaling operation with the scale factor of $\lfloor n/4\alpha \rceil$ on $ct.z_j$ for all j = 0,1,…,d. As a result, we obtain a ciphertext $ct.sum_j$ that encrypts an approximate value of $(4\alpha p/n)\Sigma_{1\le i\le n}(z_{ij}/8)$ in each plaintext slot. These ciphertexts will be stored during evaluation and used for updating the j-th component of weight vector β. In particular, the ciphertexts $ct.beta_0,\ldots,ct.beta_d$ corresponding to the entries of β become $ct.sum_0,\ldots,ct.sum_d$ at the first iteration.

Figure S1 shows how to evaluate the arithmetic circuit $(2\cdot g(z_i^{\mathrm{T}}\beta) - 1)\cdot(z_i/8)$ when $g(x) = g_3(x)$ or $g(x) = g_7(x)$. We take encryptions of $p\cdot\beta$ and $p\cdot(z_i/8)$ as inputs of the algorithm to minimize the number of required multiplications and depth. Consequently, the proposed method reduces the ciphertext modulus by 3·log p + ⌈log($n/4\alpha$)⌉ bits or 4·log p + ⌈log($n/4\alpha$)⌉ bits when $g(x) = g_3(x)$ or $g(x) = g_7(x)$, respectively.

Figure S1. Evaluation procedure of least squares approximations $(2g(z_i^{\mathrm{T}}\beta)-1)\cdot(z_i/8)$ when $g(x)=g_3(x)$ (left) and $g(x)=g_7(x)$ (right).

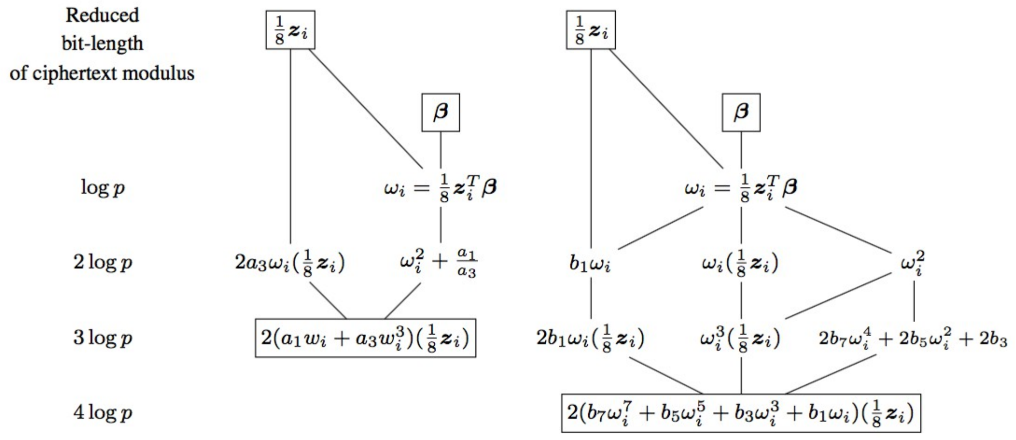

Supplement: Multimedia Appendix 2 [file medinform_v6i2e19_app2.pdf]
