# Supplementary material for: Secure Logistic Regression Based on Homomorphic Encryption: Design and Evaluation
Source: JMIR Med Inform. 2018 Apr 17;6(2):e19. doi: 10.2196/medinform.8805 (PMC5930176; doi:10.2196/medinform.8805)
Supplement: Multimedia Appendix 3 [file medinform_v6i2e19_app3.pdf]

How to Set Parameters

A lower-bound on the bit length of a fresh ciphertext modulus, denoted by log q, is as follows:

$\lceil \log(n/4\alpha) \rceil + (IterNum\text{-}1)\cdot(\lceil \log(n/4\alpha) \rceil + 3\log p) + \log q_0$ when $g(x)=g_3(x)$,

$\lceil \log(n/4\alpha) \rceil + (IterNum\text{-}1)\cdot(\lceil \log(n/4\alpha) \rceil + 4\log p) + \log q_0$ when $g(x)=g_7(x)$. (A-2)

where *IterNum* is the number of iterations of the gradient descent algorithm and q_0 is the output ciphertext modulus. The final ciphertext represents the desired vector β but is scaled by a factor of p, which means that log q_0 should be larger than log p.

The security of the underlying homomorphic encryption scheme relies on the hardness of the ring learning with errors assumption. We derive a lower bound on the ring dimension by

$N / \log Q \geq (\lambda+110) / 7.2$, (A-3)

to get λ-bit security level. In other words, we will take the smallest integer N that is a power of two satisfying this inequality (A-3).

In our implementation, each coefficient of the secret key is chosen at random from {0,1,-1} and we set the number of nonzero coefficients in the key as h=64. We used the standard deviation 3.2 for a discrete Gaussian distribution χ to sample random error polynomials. From the inequality (A-3), we took the ring dimension $N=2^{17}$ to ensure 80-bit security. For this setting, the public key and a freshly encrypted ciphertext have two ring elements in R_q so their bit size is bounded by $2N \log q \approx$ 75MB.
